# Inhibition of mitochondrial oxidative metabolism attenuates EMCV replication and protects β-cells from virally mediated lysis

**DOI:** 10.1074/jbc.RA120.014851

**Published:** 2021-01-13

**Authors:** Joshua D. Stafford, Zachary R. Shaheen, Chay Teng Yeo, John A. Corbett

**Affiliations:** Department of Biochemistry, Medical College of Wisconsin, Milwaukee, Wisconsin, USA

**Keywords:** β-cells, nitric oxide, EMCV, mitochondrial metabolism, innate immunity, autoimmune diabetes, B-cell, plus-stranded RNA virus, diabetes

## Abstract

Viral infection is one environmental factor that may contribute to the initiation of pancreatic β-cell destruction during the development of autoimmune diabetes. Picornaviruses, such as encephalomyocarditis virus (EMCV), induce a pro-inflammatory response in islets leading to local production of cytokines, such as IL-1, by resident islet leukocytes. Furthermore, IL-1 is known to stimulate β-cell expression of iNOS and production of the free radical nitric oxide. The purpose of this study was to determine whether nitric oxide contributes to the β-cell response to viral infection. We show that nitric oxide protects β-cells against virally mediated lysis by limiting EMCV replication. This protection requires low micromolar, or iNOS-derived, levels of nitric oxide. At these concentrations nitric oxide inhibits the Krebs enzyme aconitase and complex IV of the electron transport chain. Like nitric oxide, pharmacological inhibition of mitochondrial oxidative metabolism attenuates EMCV-mediated β-cell lysis by inhibiting viral replication. These findings provide novel evidence that cytokine signaling in β-cells functions to limit viral replication and subsequent β-cell lysis by attenuating mitochondrial oxidative metabolism in a nitric oxide–dependent manner.

Autoimmune diabetes is characterized by selective destruction of insulin-secreting β-cells found in pancreatic islets (1). Although genetic factors play a role in the development of this disease, the low concordance rate among monozygotic twins (40–60%) also supports a role for environmental factors as contributors to disease development (2, 3, 4). Viral infection is one environmental factor that has been associated with diabetes development due, in part, to the ability of viruses to activate innate immunity and inflammation during infection (5). Picornavirus family members, and enteroviruses in particular, have received attention as potential environmental factors that contribute to disease initiation because of their association with type 1 diabetes development in human patients (6, 7, 8). Although a direct causal relationship between enteroviral infection and type 1 diabetes development has yet to be demonstrated, studies examining the pancreases from individuals with recent-onset diabetes have found increased incidence of the enteroviral genome and viral proteins relative to nondiabetic control individuals (9). In addition, disease progression is more rapid in individuals who test seropositive for enteroviruses (10).

Encephalomyocarditis virus (EMCV) is a mouse-tropic, small, nonenveloped, positive-sense, single-stranded RNA (+ssRNA) picornavirus that induces diabetes in select mouse strains (11, 12, 13). EMCV rapidly activates pro-inflammatory signaling cascades in macrophages, leading to the local release of the inflammatory cytokines such as interleukin (IL)-1 (14). We and others have shown that treating islets with IL-1 or activating resident macrophages to release IL-1 locally in islets results in an inhibition of mitochondrial oxidative metabolism and insulin secretion that is mediated by iNOS expression and the production of micromolar levels of nitric oxide in β-cells (15, 16, 17, 18, 19). The inhibitory actions of nitric oxide on β-cell function are reversible (20, 21, 22, 23), and the recovery of function correlates with a number of protective actions that are stimulated by nitric oxide in β-cells. Specifically, we have shown that nitric oxide activates base excision DNA repair (22), simulates a protective unfolded protein response (24), and attenuates DNA damage response–mediated β-cell apoptosis (20, 21).

Nitric oxide is known to have antiviral activities that include inhibiting the replication of DNA (herpes simplex virus type 1 and vaccinia virus) and RNA (vesicular stomatitis virus) viruses, including Coxsackievirus, a picornavirus similar to EMCV (25, 26, 27, 28, 29, 30). In addition, viral clearance is attenuated and mortality is increased in mice lacking iNOS (29). We have shown that EMCV replication is elevated in mice lacking the chemokine receptor CCR5, a cell surface receptor required for EMCV to induce inflammatory gene expression in macrophages, such as iNOS (14). These findings suggest that nitric oxide may function in a protective role within the context of viral infection.

In this study, we show that nitric oxide, either supplied exogenously with donor compounds or produced endogenously in response to cytokine treatment, attenuates EMCV replication and protects β-cells against EMCV-mediated death. The antiviral actions of nitric oxide do not require new gene expression (transcription or translation) or the induction of ER stress and are restricted to concentrations in the low micromolar range (1–5 μm) or levels produced by β-cells expressing iNOS. Although previous studies have implicated *S*-nitrosation of viral proteins as a mechanism of viral inhibition (31), we provide evidence that nitric oxide protects β-cells from EMCV by inhibiting mitochondrial oxidative metabolism. Nitric oxide inhibits the Krebs cycle at aconitase (destruction of the iron-sulfur complex) and the electron transport chain at complex IV (32, 33, 34). Consistent with mitochondrial metabolism as a target of nitric oxide, we show that inhibition of mitochondrial respiration attenuates EMCV replication and protects β-cells from EMCV-mediated death. These findings support the inhibition of mitochondrial oxidative metabolism as a novel mechanism by which nitric oxide attenuates EMCV replication and protects β-cells from viral lysis.

## Results

### Nitric oxide attenuates EMCV-mediated β-cell death

**Figure 1:**
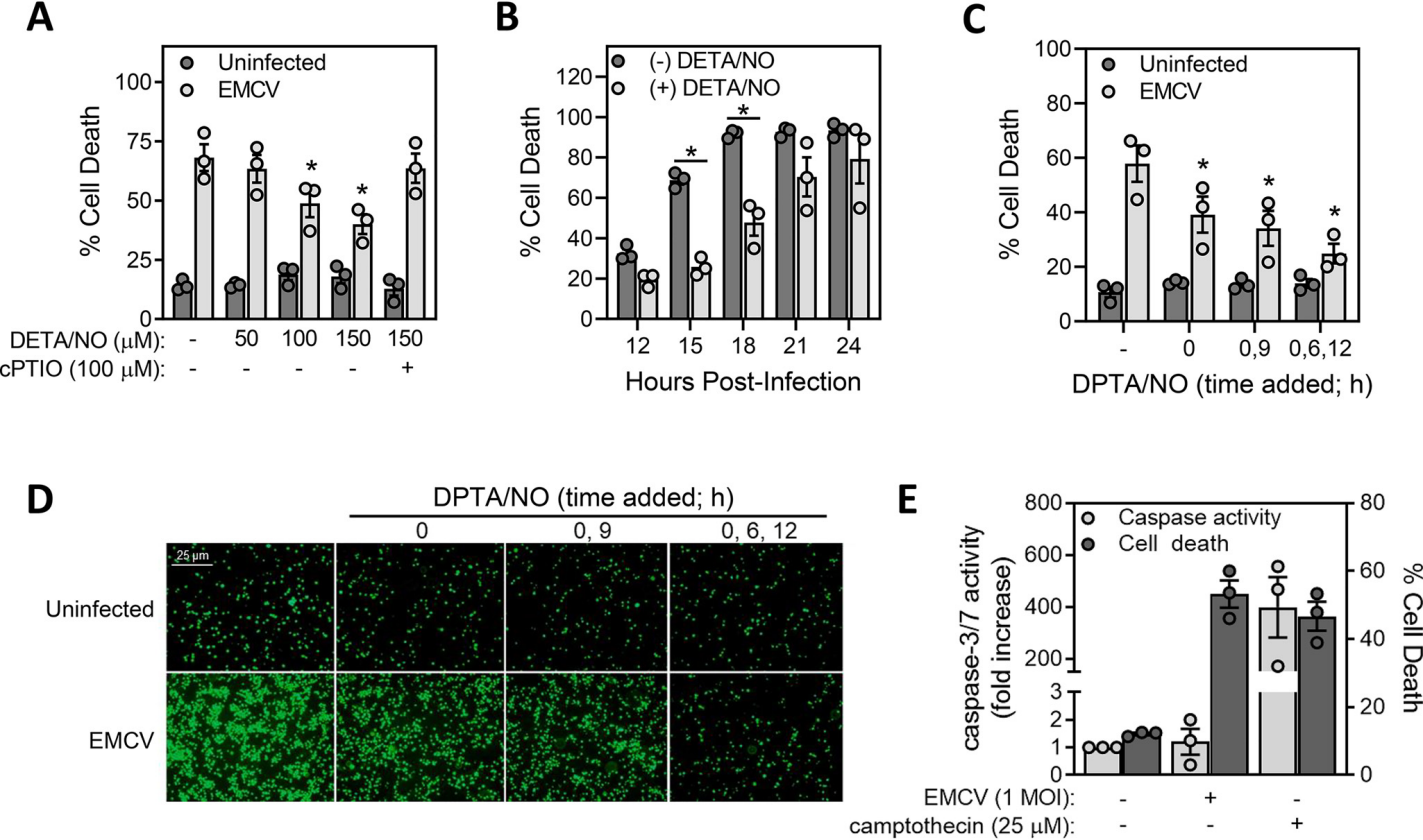
**Nitric oxide donors attenuate EMCV-mediated cell death.***A*, MIN6 cells (50,000/100 μl medium) were infected with 5 m.o.i. EMCV in the presence of increasing concentrations of DETA/NO with or without the nitric oxide scavenging agent cPTIO and cell death was measured by STYOX fluorescence 18 h post infection. *B*, MIN6 cells were infected with 5 m.o.i. EMCV with or without DETA/NO (150 μm) and cell death was measured by SYTOX fluorescence 12–24 h post infection. *C*, MIN6 cells were infected with 5 m.o.i. EMCV with or without DPTA/NO (50 μm) added at the time of infection and every 6 or 9 h post infection, as indicated. Cell death was measured by SYTOX fluorescence 18 h post infection. *D*, representative fluorescent images are shown for each condition in *panel C*. *E*, MIN6 cells (200,000/400 μl medium, caspase activity) (50,000/100 μl medium, cell death) were treated for 36 h with 1 m.o.i. EMCV or camptothecin (25 μm). Cells were lysed and caspase 3/7 activity was assessed at 24 h and cell death was measured by SYTOX fluorescence at 36 h. Results are the average ± S.E. of three independent experiments (*A*–*C*, *E*) or representative of three independent experiments (*D*). Statistically significant differences are indicated (*, *p* < 0.05).

To examine the role of nitric oxide in controlling β-cell viability in response to viral infection, murine insulinoma MIN6 cells were infected for 18 h with EMCV (5 m.o.i.) in the presence of increasing concentrations of the nitric oxide donor compound DETA/NO (*t*_1/2_ ∼20 h). Nitric oxide donors were used because many mouse insulinoma cell lines, such as MIN6 cells, do not express iNOS in response to cytokines (data not shown). Also, iNOS expression in murine β-cells requires a combination of IL-1 and IFN-γ (35), and IFN-γ is known to have antiviral activities that could mask the antiviral actions of nitric oxide. In a concentration-dependent manner DETA/NO attenuates EMCV-mediated lysis of MIN6 cells (Fig. 1*A*). The nitric oxide scavenger cPTIO attenuates the protective actions of DETA/NO, supporting nitric oxide or nitric oxide–derived products as the chemical species that protects β-cells from EMCV-mediated lysis. The protective actions of DETA/NO (150 μm) on EMCV-mediated cell lysis are time-dependent, with maximal protection observed early in the treatment (12, 15 h) and this protection is lost following longer exposures (20 h or greater) (Fig. 1*B*).

We hypothesized that the loss of protection is because of degradation of DETA/NO such that the concentration of nitric oxide that is released falls below a threshold level, or a level that is consistent with the amount of nitric oxide produced endogenously by iNOS. This hypothesis was confirmed using a multiple addition approach with a second nitric oxide donor DPTA/NO (*t*_1/2_ ∼3 h). As shown in Fig. 1*C*, the addition of DPTA/NO during infection and every 6 h post infection provides almost complete protection against EMCV-mediated MIN6 cell death (Fig. 1, *C* and *D*). The protection afforded by nitric oxide is decreased when DPTA/NO is added only during and 9 h post infection, and nitric oxide is least effective when supplied only at the time of infection (Fig. 1, *C* and *D*). These data suggest that sustained production of nitric oxide in the low micromolar (or iNOS-derived) range protects β-cells from EMCV and that the protective actions are lost when nitric oxide is no longer generated at this level.

Because nitric oxide is an effective inhibitor of caspase activity and apoptosis (36, 37, 38) and nitric oxide attenuates EMCV-mediated MIN6 cell death, the effects of EMCV infection on caspase activation were examined. Consistent with EMCV being a lytic virus that encodes proteins that inhibit apoptosis (13, 39), infection of MIN6 cells fails to stimulate caspase 3 or 7 activity (Fig. 1*E*). As a control for apoptosis induction, we show that the topoisomerase inhibitor camptothecin stimulates robust caspase 3 and 7 activity in MIN6 cells. Overall, these findings are consistent with EMCV being a lytic virus that induces cell necrosis and indicate that nitric oxide protects these cells by a mechanism that differs from its ability to attenuate caspase-dependent insulinoma cell death.

### Role of new gene transcription and type I IFN production in protection from EMCV-mediated β-cell lysis

**Figure 2:**
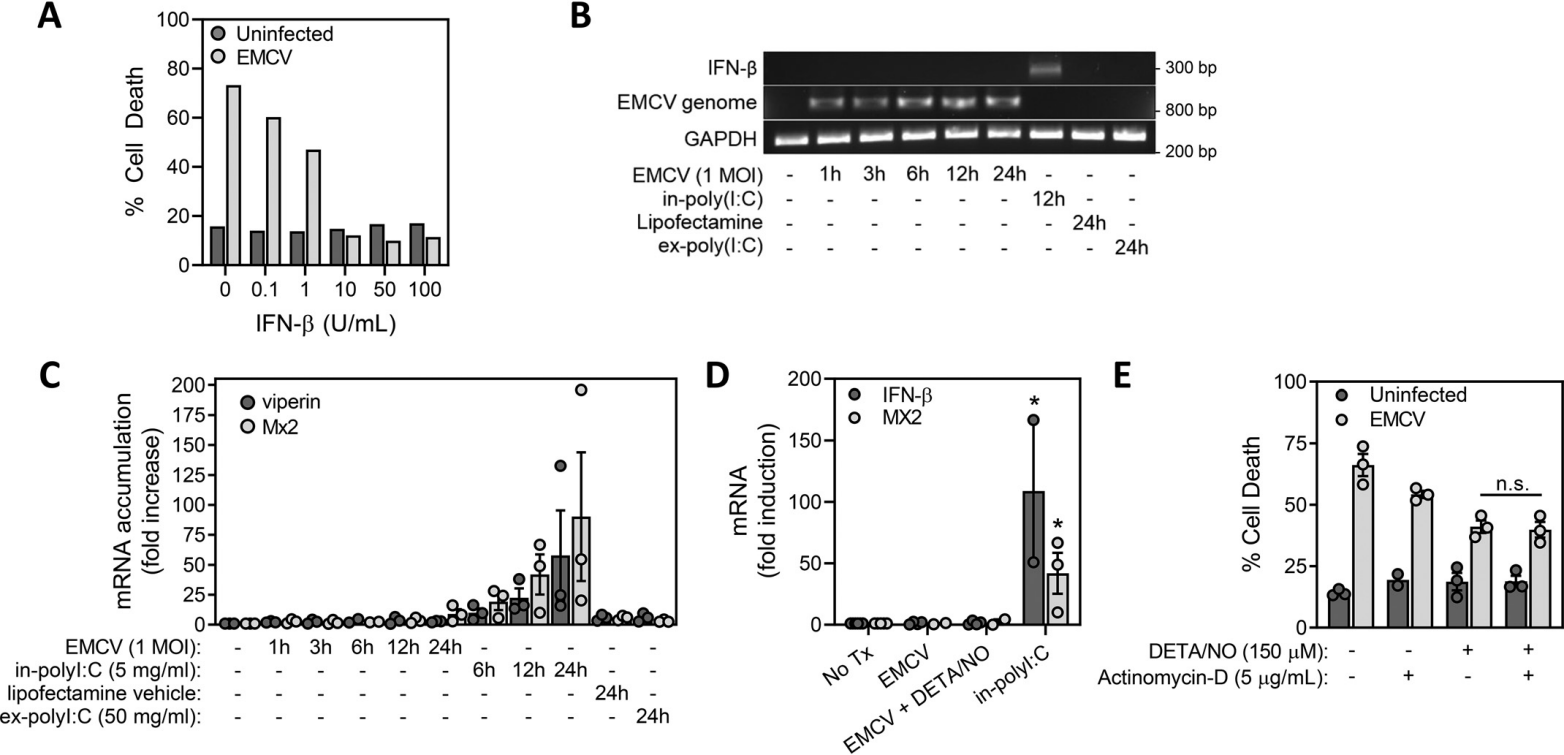
**The protective actions of nitric oxide are independent of IFN expression or new gene transcription.***A*, MIN6 cells (50,000/100 μl medium) were infected with 5 m.o.i. EMCV and treated with increasing concentrations of IFN-β and cell death was measured by SYTOX fluorescence 18 h post infection. *B* and *C*, MIN6 cells (200,000/400 μl medium) were treated with 1 m.o.i. EMCV, intracellular polyI:C (5 µg/ml), lipofectamine vehicle, or extracellular polyI:C (50 µg/ml) for up to 24 h, and EMCV and IFN-β mRNA (*B*) and ISG mRNA (*C*) accumulation were determined by qRT-PCR and agarose gel electrophoresis. *D*, MIN6 cells were infected with EMCV (5 m.o.i.) in the presence or absence of DETA/NO (150 μm) or were treated with polyI:C (5 µg/ml) complexed with lipofectamine for 12 h and IFN-β and MX2 mRNA accumulation was determined by qRT-PCR. *E*, MIN6 cells (50,000/100 μl medium, in triplicate) were infected with 5 m.o.i. EMCV and treated with DETA/NO (150 μm) or actinomycin-D (5 µg/ml), and cell death was measured by STYOX fluorescence 18 h post infection. Results are representative (*B*) or the average ± S.E. of three independent experiments (*C*–*E*). Statistically significant differences are indicated (*, *p* < 0.05).

The classic cellular response to viral infection is the induction of type I interferons (IFNs), which then induce the expression of >500 antiviral genes (40, 41). Consistent with the protective actions of type I IFN, EMCV-mediated MIN6 cell death is attenuated in a concentration-dependent manner by IFN-β (Fig. 2*A*); however, EMCV fails to stimulate expression of type I IFN (Fig. 2*B*) or the expression of MX2 or viperin, two interferon-stimulated genes (ISGs) (Fig. 2*C*). The absence of type I IFN expression, even though EMCV mRNA accumulates in infected cells (Fig. 2*B*), is consistent with the suppression of interferon expression by the EMCV-encoded Leader protein, which circumvents host antiviral defenses by impairing trafficking between the nucleus and cytoplasm (42, 43, 44). Further, the type I IFN response is not activated under conditions in which nitric oxide affords protection against EMCV, as mRNA expression of IFN-β and the interferon-stimulated gene MX2 are not induced in MIN6 cells infected with EMCV in the presence or absence of 150 μm DETA/NO (Fig. 2*D*). As a positive control for these studies, MIN6 cells were transfected with the synthetic dsRNA compound polyinosinic:polycytidylic acid (polyI:C), which induces expression of both IFN-β and ISGs (Fig. 2, *B*–*D*) (45). The protective actions of nitric oxide also do not require *de novo* gene expression as the host transcription inhibitor actinomycin D does not modify the protective actions of DETA/NO on EMCV-mediated MIN6 lysis (Fig. 2*E*). These findings disassociate *de novo* gene expression and the induction of classical type I antiviral IFN responses from the protective actions of nitric oxide on EMCV-mediated β-cell lysis.

### EMCV replication is attenuated in the presence of nitric oxide

**Figure 3:**
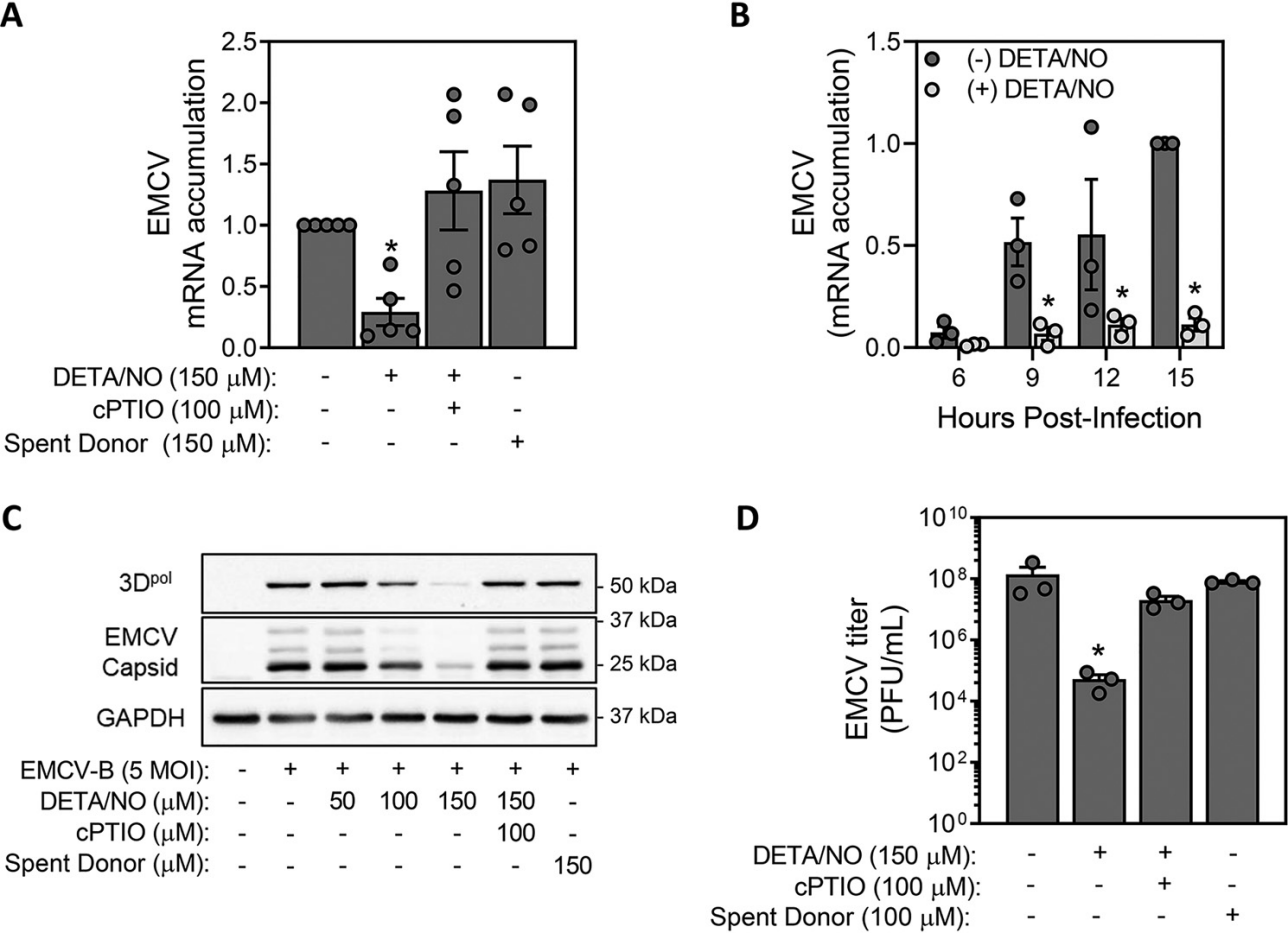
**EMCV replication is attenuated in the presence of nitric oxide.***A* and *B*, EMCV mRNA (VP1) accumulation was determined by qRT-PCR in MIN6 cells (200,000/400 μl medium) infected with 5 m.o.i. EMCV for 12 h with or without DETA/NO, cPTIO, or spent donor controls (*A*), or the time-dependent effects of DETA/NO were examined (*B*). *C* and *D*, accumulation of viral polymerase (3D^pol^) and capsid protein was determined by Western blotting 18 h post infection (*C*) and EMCV titers were determined from the supernatant by plaque assay at 24 h post infection (*D*). Results are the average ± S.E. of three to five independent experiments (*A*, *B*, and *D*) or representative (*C*) of three independent experiments. Statistically significant differences are indicated (*, *p* < 0.05).

We have shown that the chemokine receptor CCR5 is required for macrophage production of nitric oxide in response to EMCV infection (14), and in mice deficient in CCR5, EMCV replicates to levels that are 11-fold higher than those in WT control mice (14). These findings suggest that nitric oxide may attenuate EMCV replication in β-cells. Consistent with this hypothesis, we show that DETA/NO attenuates EMCV mRNA accumulation in MIN6 cells in a nitric oxide–dependent manner as scavenging with cPTIO attenuates this response (Fig. 3*A*). Also, donor that has liberated its nitric oxide (spent donor) does not inhibit EMCV mRNA accumulation (Fig. 3*A*). The inhibitory actions of nitric oxide occur at all time points examined between 6 and 15 h post infection (Fig. 3, *A* and *B*). This nitric oxide donor also attenuates the accumulation of RNA-dependent RNA polymerase (3D^pol^) and three subunits of the viral capsid in a concentration-dependent manner as measured by Western blot analysis at 18 h post infection (Fig. 3*C*). Spent donor has no effect, whereas cPTIO attenuates the inhibitory actions of nitric oxide on EMCV 3D^pol^ and capsid protein expression (Fig. 3*C*). Consistent with the inhibition of EMCV mRNA and protein expression, DETA/NO treatment leads to a 3-log decrease in the formation of infectious virions as determined by plaque assay (Fig. 3*D*). These data support the inhibition of viral replication as one mechanism by which nitric oxide limits EMCV-mediated MIN6 cell lysis.

### The antiviral actions of nitric oxide are not mediated by the induction of ER stress

**Figure 4:**
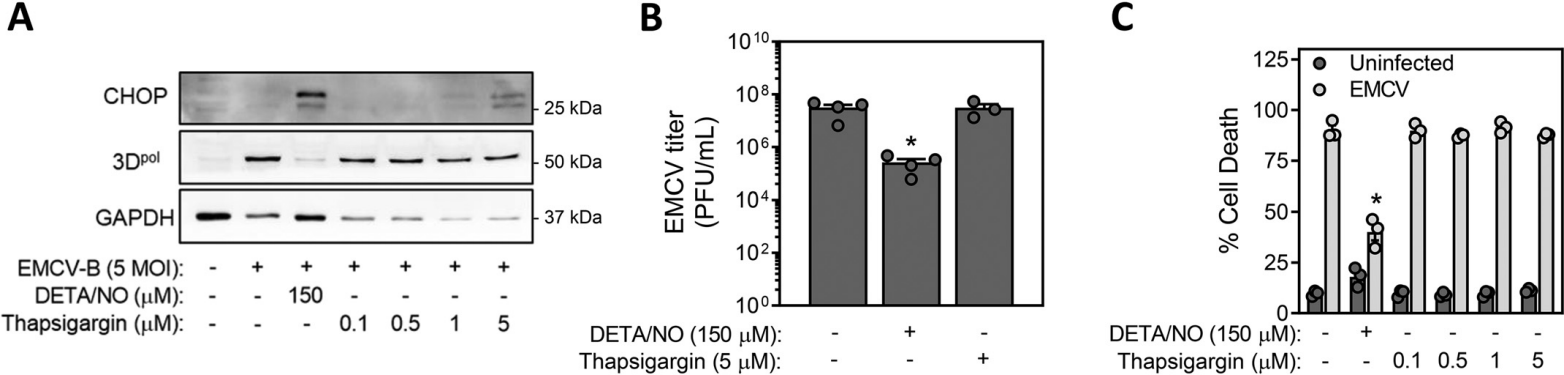
**The protective actions of nitric oxide against EMCV are not mediated by the induction of ER stress.***A*–*C*, MIN6 cells (200,000/400 μl medium) were infected with EMCV (5 m.o.i.) and were treated with DETA/NO (150 μm) or increasing concentrations of thapsigargin for 18 h. Accumulation of EMCV protein (3D^pol^) and ER stress markers (CHOP) were determined by Western blotting (*A*), EMCV titers were determined from the supernatant by plaque assay (*B*), and cell death was measured via SYTOX assay (*C*). Results are the average ± S.E. of three independent experiments (*B* and *C*) or representative (*A*) of three independent experiments. Statistically significant differences are indicated (*, *p* < 0.05).

Nitric oxide stimulates unfolded protein response (UPR) activation through the inhibition of the calcium ion pump sarco(endo)plasmic reticulum Ca^2+^-ATPase (SERCA). Once activated, the UPR transducer protein kinase R-like ER kinase (PERK) phosphorylates and inhibits eukaryotic initiation factor (eIF)2α, thereby inhibiting cap-dependent translation (46, 47, 48). We used the SERCA inhibitor thapsigargin to examine whether UPR activation contributes to the inhibitory actions of nitric oxide on EMCV replication. As shown in Fig. 4*A*, both DETA/NO and thapsigargin stimulate ER stress activation, as evidenced by CHOP accumulation; however, only nitric oxide inhibits the accumulation of EMCV polymerase expression (Fig. 4*A*). Consistent with these findings, thapsigargin does not modify EMCV-mediated MIN6 cell lysis or EMCV titer whereas nitric oxide attenuates the loss of cell viability (Fig. 4*B*) and decreases viral titer (Fig. 4*C*). Even though UPR activation results in an attenuation in translation, our findings are consistent with a requirement for EMCV to use host translational machinery through an internal ribosomal entry site (IRES) that is cap-independent (13, 49). These studies also indicate that the inhibitory actions of nitric oxide on EMCV replication are mediated by pathways independent of UPR activation.

### Inhibition of oxidative metabolism protects β-cells from EMCV

Nitric oxide is known to target and inhibit mitochondrial metabolism, through inhibition of aconitase and complexes I and IV of the electron transport chain (32, 33, 34, 50). The net effect of this inhibition of mitochondrial oxidative metabolism is a decrease in islet ATP levels (51). In fact, it is through the inhibition of oxidative metabolism that nitric oxide mediates the inhibitory actions of cytokines on insulin secretion from islets of all species examined to date (17).

**Figure 5:**
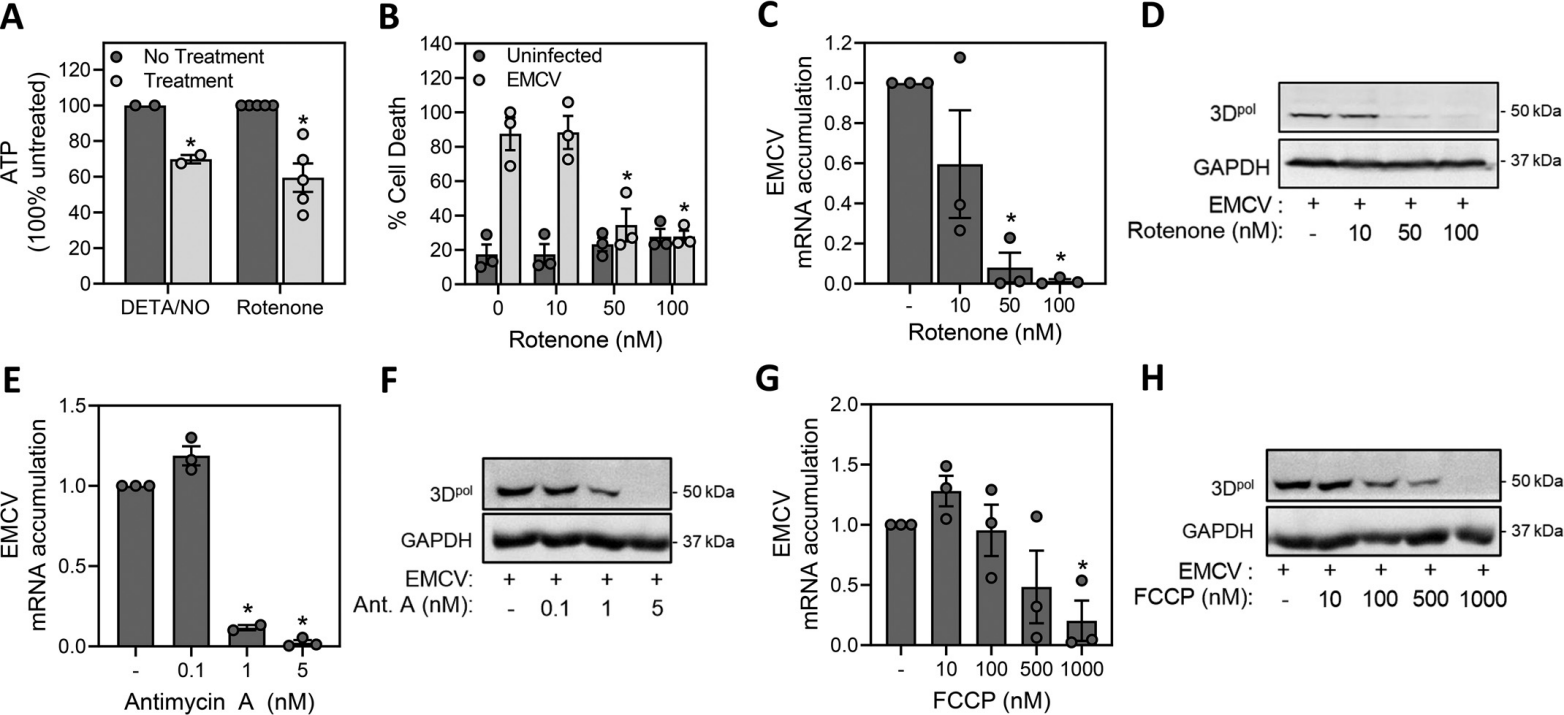
**Inhibition of mitochondrial oxidative metabolism attenuates EMCV replication in** β**-cells.***A*, cellular levels of ATP were determined by HPLC analysis from MIN6 cells treated for 2 h with DETA/NO (150 μm) or rotenone (75 nm). ATP levels were normalized to total protein concentrations. *B*, the viability of MIN6 cells (50,000 cells/100 μl medium) infected with EMCV (5 m.o.i.) and treated with increasing concentrations of rotenone for 18 h was measured via SYTOX fluorescence. *C*, *E*, and *G*, accumulation of EMCV mRNA in infected MIN6 cells (5 m.o.i.) was measured by qRT-PCR 12 h post infection. *D*, *F*, and *H*, accumulation of EMCV protein was measured by Western blotting 24 h post infection. Results are the average ± S.E. of two to five independent experiments (*A*–*C*, *E*, *G*) or representative (*D*, *F*, *H*) of three independent experiments. Statistically significant differences are indicated (*, *p* < 0.05).

The inhibition of oxidative metabolism also appears to be the mechanisms by which nitric oxide attenuates EMCV replication in β-cells. Like nitric oxide (DETA/NO, 150 μm), the electron transport chain complex I inhibitor rotenone (75 nm) decreases the level of ATP and attenuates the loss in the viability of MIN6 cells infected with EMCV (5*A* and *B*). In a similar concentration-dependent manner rotenone attenuates EMCV mRNA accumulation and 3D^pol^ expression (Fig. 5, *C* and *D*). Consistent with the actions of nitric oxide and rotenone, the complex III inhibitor antimycin A and the uncoupling agent FCCP also attenuate EMCV mRNA and protein accumulation (Fig. 5, *E*–*H*). These findings support the inhibition of mitochondrial oxidative metabolism as one mechanism by which nitric oxide attenuates EMCV replication.

### Nitric oxide and rotenone attenuate EMCV replication in islet cells

**Figure 6:**
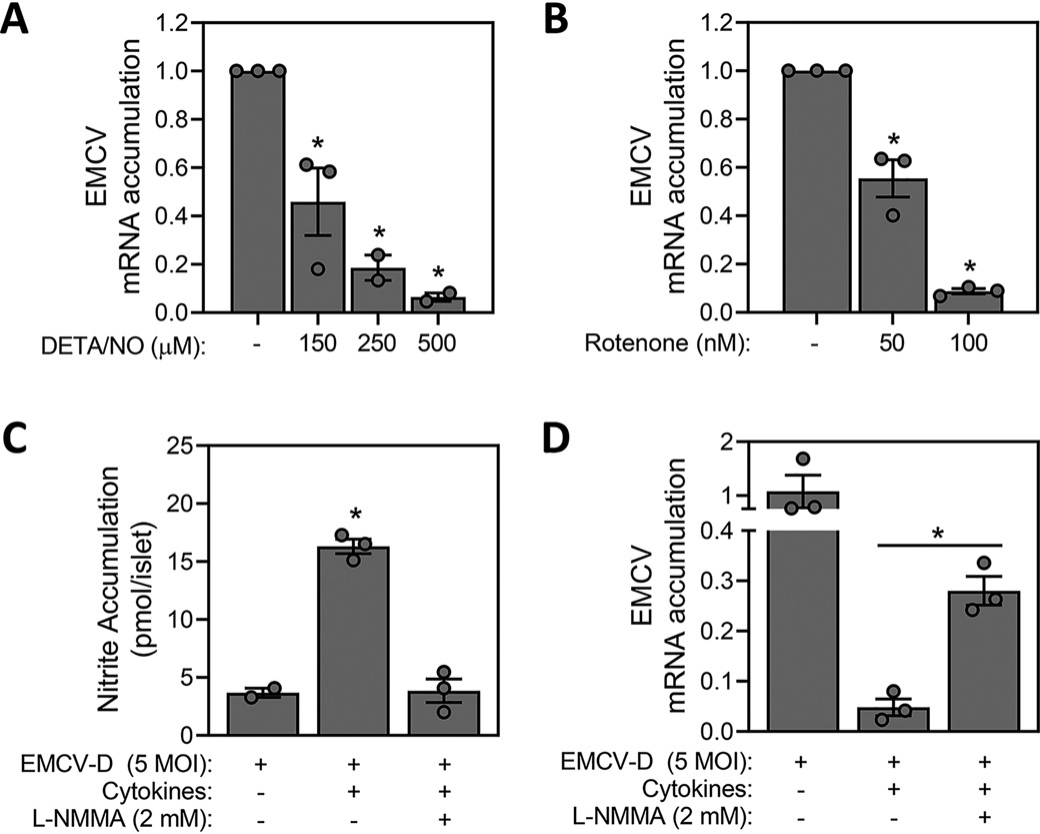
**Nitric oxide attenuates EMCV replication in mouse islets.***A* and *B*, islets were isolated from C57BL/6 mice and dispersed into individual cells prior to EMCV infection (5 m.o.i.) in the presence of DETA/NO (*A*) or rotenone (*B*). EMCV mRNA accumulation was measured by qRT-PCR 12 h post infection. Intact DBA/2J islets were treated with IL-1β (10 units/ml) and IFN-γ (15 units/ml) in the presence or absence of l-NMMA (2 mm) and infected with EMCV (5 m.o.i.). *C* and *D*, at 24 h post infection, nitrite (*C*) and EMCV mRNA (*D*) were measured by Griess assay and qRT-PCR, respectively. Results are the average ± S.E. of three independent experiments. Statistically significant differences are indicated (*, *p* < 0.05).

Consistent with the inhibition of viral replication in insulinoma cell lines, nitric oxide and rotenone attenuate EMCV replication in mouse islets. For these studies, islets isolated from male and female C57BL/6 mice were dispersed into individual cells and treated with DETA/NO (Fig. 6*A*) or rotenone (Fig. 6*B*) prior to EMCV infection. In a concentration-dependent manner, both DETA/NO and rotenone attenuate the accumulation of EMCV mRNA, indicating that the inhibition of mitochondrial oxidation attenuates the replication of EMCV in islet cells.

Because β-cells produce micromolar levels of nitric oxide in response to cytokines (35), we examined whether the endogenous production of nitric oxide would limit EMCV replication in islet cells. Complicating these studies is the requirement of IFN-γ, a cytokine known to have antiviral activity, along with IL-1β to induce expression of iNOS and production of nitric oxide by β-cells. To explore the role of endogenous production of nitric oxide in viral replication, islets isolated from DBA/2J mice (a strain that is susceptible to EMCV-induced diabetes (11, 12)) were treated with IL-1β and IFN-γ and then infected with 5 m.o.i. EMCV in the presence or absence of an iNOS inhibitor l-NMMA. In response to cytokine treatment DBA/2J mouse islet cells produce nitric oxide and this is attenuated by l-NMMA (Fig. 6*C*). As expected, treatment with the antiviral cytokine IFN-γ inhibits EMCV replication even in the presence of l-NMMA; however, l-NMMA attenuates the inhibitory actions of IL-1 + IFN-γ on EMCV replication indicating that nitric oxide also has antiviral properties in β-cells (Fig. 6*D*). These findings show that nitric oxide, when produced endogenously by β-cells following cytokine stimulation, functions as an antiviral agent to limit EMCV replication.

## Discussion

It has long been known that inhibitory actions of cytokines on insulin secretion are mediated by β-cell production of nitric oxide (15, 16, 17, 18). We and others have shown that inhibitors of NOS prevent cytokine-induced inhibition of insulin secretion from rodent and human islets (52, 53, 54). The mechanism by which nitric oxide attenuates insulin secretion is via the inhibition of the Krebs cycle enzyme aconitase and complex IV of the electron transport chain (32, 33, 34, 50, 51) resulting in a decrease in β-cell ATP levels (32, 51). Because increases in ATP (ATP/ADP ratio) are required to close K^+^_ATP_ channels for glucose-stimulated insulin secretion (55), it is this decrease in ATP that results from the inhibition of mitochondrial respiration that is the primary mechanism by which nitric oxide impairs insulin secretion by β-cells. Nitric oxide also induces DNA damage, and it is this combination of DNA damage and impaired mitochondrial oxidation that is believed to be responsible for the loss of β-cell viability in response to cytokine treatment (19, 35, 56). Whether nitric oxide reduces β-cell viability by necrosis or apoptosis has been controversial (53), however, nitric oxide is an effective inhibitor of apoptotic signaling and caspase activation (20, 21, 36, 37, 38). Further, it is challenging to induce apoptosis in primary β-cells by any means (57, 58).

Many studies have shown that nitric oxide mediates the inhibitory actions of cytokines on insulin secretion and these actions have been interpreted as being damaging to β-cells (22, 52, 53, 59). In this study, we provide evidence in support of the contrasting view that β-cell production of nitric oxide in response to cytokines functions in a physiologically relevant protective role as part of a mechanism to limit viral replication and β-cell lysis during infection. We show that nitric oxide is an effective inhibitor of viral replication thereby protecting β-cells from the rapidly lytic encephalomyocarditis virus. The mechanism through which nitric oxide attenuates EMCV-mediated β-cell lysis (Fig. 1) is distinct from classical antiviral recognition, namely, the induction of type I IFNs (IFN-α and IFN-β) and the expression of antiviral genes (40, 41). Although dsRNA, produced as an intermediate during viral replication, is recognized by host dsRNA sensors leading to the induction of the type I IFN response (45, 60, 61), EMCV circumvents this response by expressing the Leader protein. This protein inhibits cytosolic dsRNA sensor antiviral responses by binding to and inhibiting Ran-GTPase and thereby preventing the nuclear translocation of transcription factors required for IFN expression (42, 43, 44). We show that EMCV fails to stimulate type I IFN production whether in the presence or absence of nitric oxide (Fig. 2), and, consistent with these observations, inhibition of host transcription using actinomycin D does not alter the protection afforded by nitric oxide against EMCV-mediated MIN6 cell death (Fig. 2).

Nitric oxide has been characterized as an antiviral molecule because of its ability to attenuate the replication of a variety of DNA and RNA viruses, and, consistent with these findings, we show that nitric oxide attenuates EMCV replication in insulinoma cells (Fig. 3) and primary mouse β-cells (Fig. 6). The first step in EMCV replication upon entry of the (+)ssRNA genome is translation of the viral polypeptide by host translational machinery (13). Although nitric oxide inhibits cap-dependent translation by activating the UPR, chemical activators of the UPR do not modify EMCV replication in MIN6 cells (Fig. 4). These findings are consistent with the translation of EMCV polypeptide through a cap-independent mechanism using an internal ribosomal entry site (13, 49). Previous studies have implicated *S*-nitrosation of viral proteins as a protective mechanism by which nitric oxide limits viral replication (31), however this is not a likely mechanism of action in β-cells. When produced at iNOS-derived levels, nitric oxide is an inhibitor of mitochondrial electron transport at complex IV and aconitase of the Krebs cycle (32), and like the actions of nitric oxide, we show that inhibitors of the electron transport chain and uncouplers of mitochondrial membrane potential attenuate EMCV replication and virally mediated lysis of MIN6 cells (Fig. 5) and mouse islets (Fig. 6). These findings support the inhibition of mitochondrial respiration as the mechanism by which nitric oxide attenuates EMCV replication. Viruses are known to reprogram host cell metabolism to provide resources for replication, establish persistence, or evade or dampen antiviral immune cell responses, and these metabolic changes vary according to the needs of each virus (62). Enteroviruses have been shown to alter host cell metabolism by enhancing the oxidation of glutamine (63), and by inhibiting mitochondrial metabolism, nitric oxide may prevent the anaplerotic utilization of this critical metabolite and thus attenuate EMCV replication.

Cytokines have long been thought to contribute to β-cell failure during the development of autoimmune diabetes because of their ability to limit oxidative metabolism, inhibit insulin secretion, and decrease β-cell viability. However, β-cells are a terminally differentiated specialized cell type that has a limited capacity to proliferate, yet they are critical for organismal survival as the only cell type that produces insulin (64). In this context, it is important to consider the possibility that the response of β-cells to cytokines serves a physiologically relevant function. β-cells are also the only islet endocrine cell that responds to cytokines with the expression of iNOS and production of nitric oxide (35, 65), further suggesting that nitric oxide functions in a physiologically relevant manner. Although many studies have attributed the inhibitory actions of cytokines to nitric oxide–dependent decreases in aconitase activity and glucose oxidation, these actions are reversible in rodent and human β-cells (52, 53, 54, 59), again suggesting a physiological role for this response. Based on data presented in this study and recent work from our laboratory (20, 21, 22, 23), we hypothesize that the physiological response of β-cells to cytokines is not damaging but instead functions as a protective response that limits damage during infection. We have shown that one protective response is the prevention of apoptosis (20), and now show that nitric oxide inhibits viral replication and β-cell lysis in response to infection with a virus from a family thought to contribute to the initiation of autoimmune diabetes (6). The mechanisms responsible for this antiviral protection include inhibition of the same pathway that controls insulin secretion in response to glucose, the nitric oxide–dependent inhibition of mitochondrial oxidative metabolism. We hypothesize that it is when this β-cell response to cytokines fails, or is not effective at limiting viral replication, that β-cell lysis occurs (66), initiating islet inflammation and the potential release of antigens that could contribute to the induction of autoimmunity directed against β-cells.

## Experimental Procedures

### Materials and animals

Male and female C57BL/6J and DBA/2J mice were purchased from The Jackson Laboratory (Bar Harbor, ME, USA) and housed in the MCW Biomedical Resource Center. All animal use and experimental procedures were approved by the Institutional Animal Care and Use Committees at the Medical College of Wisconsin.

MIN6 cells were obtained from the Washington University Tissue Culture Support Center (St. Louis, MO, USA). L929 cell lines and Eagle's Minimum Essential Medium (EMEM) were obtained from ATCC (Manassas, VA, USA). Dulbecco's modified Eagle's medium (DMEM), Connaught Medical Research Laboratories (CMRL) 1066 medium, fetal calf serum, horse serum, l-glutamine, sodium pyruvate, penicillin, streptomycin, and β-mercaptoethanol were purchased from Invitrogen. Trypsin (0.05% in 0.53 mm EDTA) was purchased from Corning (Corning, NY, USA). Human recombinant IL-1β and murine IFN-γ and IFN-β were purchased from PeproTech (Rocky Hill, NJ, USA). Thapsigargin and the nitric oxide synthase inhibitor N^G^-monomethyl l-arginine (NMMA) were purchased from Axxora (San Diego, CA, USA). Actinomycin-D, 2-(carboxyphenyl)-4,5-dihydro-4,4,5,5-tetramethyl-1H-imidazolyl-1-oxy-3-oxide monopotassium salt (cPTIO) and nitric oxide donors (Z)-1-(N-(2-aminoethyl)-N-(2-ammonioethyl)amino)diazen-1-ium-1,2-diolate (DETA/NO) and (Z)-1-(N-(3-aminopropyl)-N-(3-ammoniopropyl)amino)diazen-1-ium-1,2-diolate (DPTA/NO) were purchased from Cayman Chemical (Ann Arbor, MI, USA). Nitric oxide donor compounds were dissolved in 10 mm NaOH prior to use. Polyinosinic:polycytidylic acid (polyI:C) and rotenone were purchased from Sigma. Antibodies and sources include mouse anti-GAPDH (Invitrogen), mouse anti-CHOP (Cell Signaling Technology), rabbit anti-iNOS (Cayman Chemical), mouse anti-Mengo 3D^pol^ (Santa Cruz Biotechnology), rabbit anti-Mengo capsid (a generous gift from Dr. Ann Palmenberg, University of Wisconsin, Madison, WI, USA), and horseradish-peroxidase (HRP)-conjugated donkey anti-mouse and HRP-donkey anti-rabbit antibodies (Jackson ImmunoResearch Laboratories, West Grove, PA, USA).

### Rodent islet isolation and cell culture

Islets from adult, male and female C57BL/6J or DBA/2J mice were isolated and cultured as described previously (67, 68). Where indicated, islets were dispersed into single cells by incubation in 0.48 mm EDTA in PBS followed by disruption in 1 mg/ml trypsin in Ca^2+^/Mg^2+^-free Hank's Balanced Salt Solution prior to experimentation (69). MIN6 cells were maintained in DMEM containing 10% heat-inactivated fetal bovine serum, l-glutamine, sodium pyruvate, penicillin, and streptomycin. L929 cells were maintained in EMEM containing 10% heat-inactivated horse serum. Both MIN6 and L929 cells were incubated at 37°C under an atmosphere of 5% CO_2_ for at least 6 h prior to the initiation of experiments. MIN6 and L929 cells were removed from growth flasks by treatment with 0.05% trypsin in 0.53 mm EDTA at 37°C for 5 min, washed twice, and plated at the indicated concentrations.

### EMCV propagation and infection

The B and D variants of EMCV were a generous gift from Dr. Ji-Won Yoon (University of Calgary, Calgary, Alberta, Canada) and have been described previously (70). EMCV was propagated in L929 cells, supernatants were clarified by centrifugation, and viral titers were determined by plaque assay. Cell monolayers were infected by the indicated multiplicity of infection (m.o.i.) for 1 h at 37°C prior to washing and replacing of media for continued culture for the indicated times. Insulinoma cells were infected with EMCV-B and mouse islet cells were infected with EMCV-D.

### Plaque assay

L929 cells were plated at 100,000 cells/ml in a 12-well tissue culture-treated plate and 150 μl of serially diluted supernatant was added to each well. Following a 30-min incubation at 37°C, viral overlay media (1% carboxymethylcellulose, 5% FBS in DMEM) was added to each well followed by continued culture for 2 days. Wells were stained and fixed with crystal violet and viral titer was calculated by counting plaques.

### Cell death assay

Cell death was determined using the SYTOX Green nucleic acid stain (Invitrogen) as described previously (20), and fluorescent images of SYTOX staining were captured using a Nikon TI-U inverted microscope.

### Nitrite determination

Nitric oxide production was assessed by measuring the accumulation of its stable metabolite nitrite in culture supernatant using the Griess Assay (71).

### Real time PCR

Total RNA was purified from cell lysates using the RNeasy Mini Kit (Qiagen) according to manufacturer's instructions. DNase digestion was performed using Turbo DNA-free procedure (Applied Biosystems) and first-strand cDNA synthesis was performed using the oligo(dT) and reverse transcriptase Superscript Preamplification System (Invitrogen) per manufacturer's instructions. Quantitative real-time PCR was performed using SsoFast Evagreen Supermix (Bio-Rad) and the Bio-Rad CFX96 Real-Time detection system per manufacturer's instructions. Each sample was normalized to GAPDH (ΔCT) and expressed as a -fold change relative to controls via the ΔΔCT method. Primers purchased from Integrated DNA Technologies and sequences were as follows: 5′-GACATCAAGAAGGTGGTGAAGC-3′ and 5′-TCCAGGGTTTCTTACTCCTTGG-3′ for GAPDH; 5′-GCACTGGGTGGAATGAGACTATTG-3′ and 5′-TCTGAGGCATCAACTGACAGGTC-3′ for IFN-β; 5′-ACAGGGGTGAATACTTGGGC-3′ and 5′-TGAAAGCCACCTTGTAATCCCT-3′ for viperin; 5′-GCTTTTAACCAGGACATCACTGC-3′ and 5′-AGTTTGGACTTGGTAGTTCTGTG-3′ for MX2; 5′-ACCTGCTTGGCCATCCTTTC-3′ and 5′-CCGAATAAACTTCAGCAGATCACC-3′ for RNaseL; 5′-GGAGTTGAGAATGCTGAGAG-3′ and 5′-TCCAGGGTTTCTTACTCCTTGG-3′ for VP1.

### Western blot analysis

Cells and islets were washed with PBS, lysed in Laemmli buffer, proteins were separated by SDS gel electrophoresis, and Western blot analysis was conducted as described previously (72). The following dilutions of primary and secondary antibodies were used: mouse anti-GAPDH, 1:10,000; mouse anti-CHOP, rabbit anti-iNOS, and rabbit anti-Mengo, 1:1000; mouse anti-3B7^pol^ 1:500; donkey anti-mouse antibody-horseradish peroxidase and donkey anti-rabbit antibody-horseradish peroxidase, 1:20,000. Immunoreactivity was detected using chemiluminescence (73).

### ATP measurement

Nucleotide levels (ATP) were quantified using HPLC as described previously (74, 75). In brief, nucleotides were extracted by perchloric acid precipitation and diluted in solvent A (100 mm potassium phosphate and 4 mm tetrabutylammonium bisulfate, pH 6.0, diluted with 20% methanol at 64:36, v/v) and analyzed by HPLC using a SUPELCOSIL LC-18-T column (3 μm, 150 × 4.6-mm internal diameter). Protein concentration was determined using the Thermo Scientific Pierce BCA Protein Assay Kit from the extraction pellet. Nucleotides were then normalized to total protein and expressed in nanomoles per milligram of protein.

### Statistics

Statistical comparisons between conditions were made using a two-tailed unpaired *t* test or between groups using either one- or two-way analysis of variance (ANOVA). Significant differences between groups were determined using the Tukey-Kramer post hoc test or Dunnett's multiple comparisons test. Statistically significant differences (*p* < 0.05) are indicated with an asterisk (*).

## Data availability

All of the data are in the manuscript.
